# Avian influenza H9N2 virus isolated from air samples in LPMs in Jiangxi, China

**DOI:** 10.1186/s12985-017-0800-y

**Published:** 2017-07-24

**Authors:** Xiaoxu Zeng, Mingbin Liu, Heng Zhang, Jingwen Wu, Xiang Zhao, Wenbing Chen, Lei Yang, Fenglan He, Guoyin Fan, Dayan Wang, Haiying Chen, Yuelong Shu

**Affiliations:** 1 Chinese National Influenza Center, National Institute for Viral Disease Control and Prevention, Collaboration Innovation Center for Diagnosis and Treatment of Infectious Diseases, Chinese Center for Disease Control and Prevention, Key Laboratory for Medical Virology, National Health and Family Planning Commission, 155 Changbai Road, Beijing, 102206 People’s Republic of China; 2 Nanchang Center for Disease Control and Prevention, Nanchang city, 330038 People’s Republic of China

**Keywords:** Influenza, Type a, H9N2, Aerosol, Phylogenetic, Transmission

## Abstract

**Background:**

Recently, avian influenza virus has caused repeated worldwide outbreaks in humans. Live Poultry Markets (LPMs) play an important role in the circulation and reassortment of novel Avian Influenza Virus (AIVs). Aerosol transmission is one of the most important pathways for influenza virus to spread among poultry, from poultry to mammals, and among mammals.

**Methods:**

In this study, air samples were collected from LPMs in Nanchang city between April 2014 and March 2015 to investigate possible aerosol transmission of AIVs. Air samples were detected for Flu A by Real-Time Reverse Transcription-Polymerase Chain Reaction (RRT-PCR). If samples were positive for Flu A, they were inoculated into 9- to 10-day-old specific-pathogen-free embryonated eggs. If the result was positive, the whole genome of the virus was sequenced by MiSeq. Phylogenetic trees of all 8 segments were constructed using MEGA 6.05 software.

**Results:**

To investigate the possible aerosol transmission of AIVs, 807 air samples were collected from LPMs in Nanchang city between April 2014 and March 2015. Based on RRT-PCR results, 275 samples (34.1%) were Flu A positive, and one virus was successfully isolated with embryonated eggs. The virus shared high nucleotide homology with H9N2 AIVs from South China.

**Conclusions:**

Our study provides further evidence that the air in LPMs can be contaminated by influenza viruses and their nucleic acids, and this should be considered when choosing and evaluating disinfection strategies in LPMs, such as regular air disinfection. Aerosolized viruses such as the H9N2 virus detected in this study can increase the risk of human infection when people are exposed in LPMs.

**Electronic supplementary material:**

The online version of this article (doi:10.1186/s12985-017-0800-y) contains supplementary material, which is available to authorized users.

## Background

While aquatic birds are the natural hosts of avian influenza virus (AIVs), the transmission of AIVs from wild birds to poultry has been seen in many places in the world, and the virus has caused frequent sporadic human outbreaks owing to contacts between poultry and humans [1–6]. During the past 20 years, live poultry markets (LPMs), which are widely distributed in China and other developing countries, were proved to be the major source of human infections with AIVs, as well as a place for potential AIV reassortment and interspecies transmission [7, 8].

In previous studies searching for the source of human infection in LPMs, close attention was paid to poultry throat and cloaca swabs, as well as environmental samples such as feces, feathers, and poultry drinking water, while aerosol samples in LPMs were poorly investigated. Aerosol transmission is one of the most important pathways for influenza virus to spread among poultry, from poultry to mammals, and among mammals [9–11]. In recent years, human infection with many subtypes of AIVs has been reported, including H5N1, H5N6, H7N9, H10N8, H6N1, and H9N2 AIVs, and field epidemiological investigation showed some patients had no history of direct contact with any live poultry before illness onset [3, 12, 13].For example, in late 2013, the first human infection with avian influenza H10N8 virus was reported in Nanchang city, Jiangxi province, China. The patient had visited the market 4 days before the onset of disease without touching any poultry [2]. To study possible aerosol transmission of AIVs in LPMs, air samples were collected from LPMs in Nanchang city between April 2014 and March 2015.

## Methods

### Sample collection

**Fig. 1:**
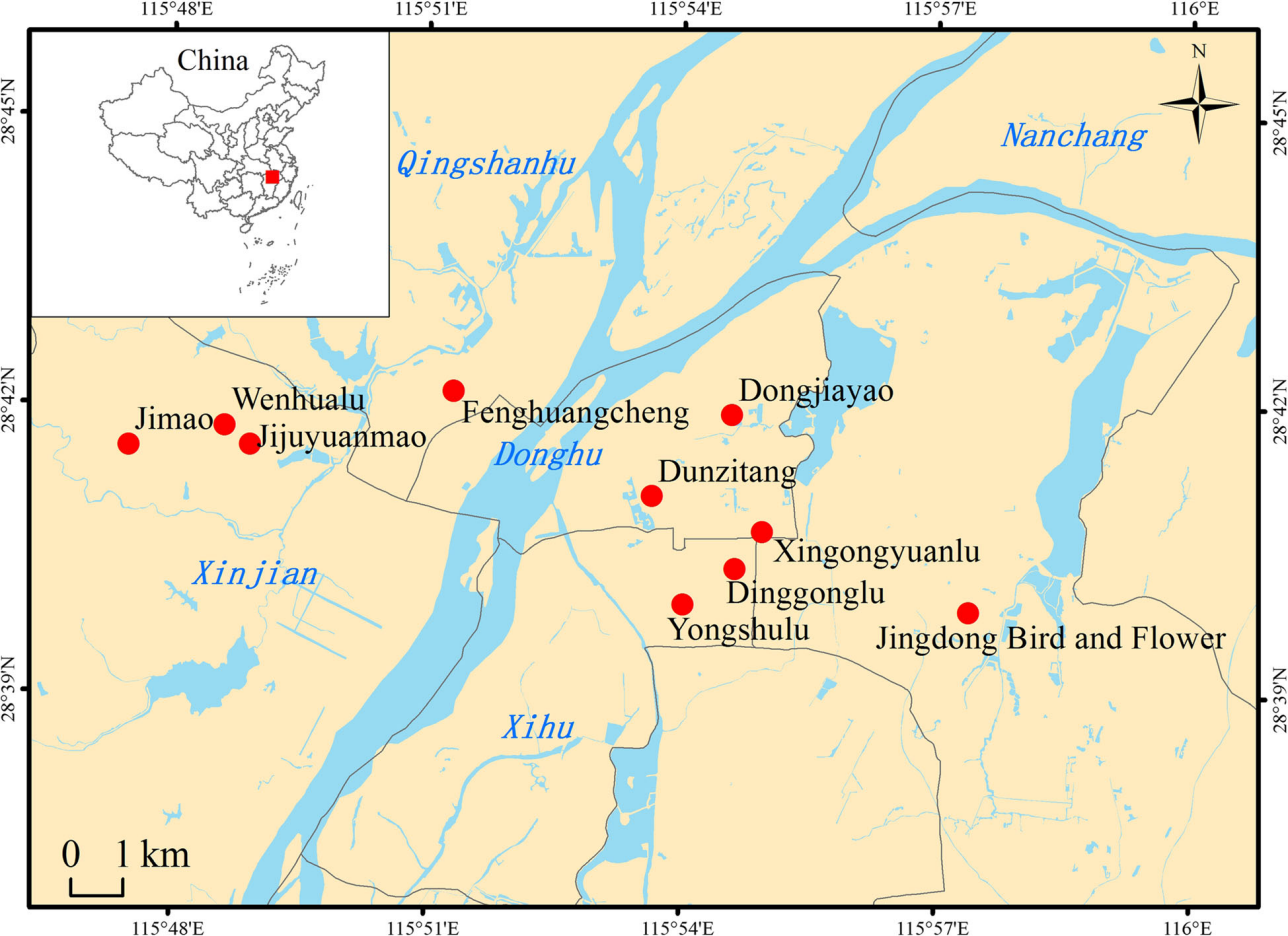
Map of ten live poultry markets in Nanchang city, Jiangxi province, China. Geographical locations of ten live poultry markets in four districts in Nanchang city, Jiangxi province, China. Jimao, Wenhualu, Jijuyuanmao, Fenghuangcheng, Dongjiayao, Dunzitang, Xingoyuanlu, Dinggonglu, Yongshulu, and Jingdong Bird and Flower market are shown by *red dots*. The map was created in ArcGIS version 10.2 software (ESRI)

**Fig. 2:**
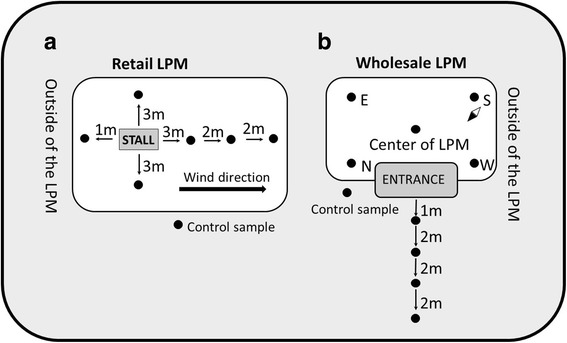
Map of air sample collection sites. **a** Retail LPMs: several poultry stalls in each retail LPM were selected as study centers. Sampling sites around the poultry stall are shown by *black dots*. Control samples were collected outside of each market. **b** Wholesale LPM: for Jingdong Bird and Flower wholesale market, five air samples were collected in the east, south, west, north, and center of the LPM, and a series of samples were collected near the outside entrance at distances of 1 m, 3 m, 5 m, and 7 m. Sampling sites are shown by *black dots*. Control sample was collected outside of the market

In this study, 46 sample sites in ten different LPMs were selected in Nanchang city, Jiangxi province, China. These LPMs were distributed in four districts (Xinjian, Donghu, Xihu, and Qingshanhu) (Fig. 1) of the city. Jingdong Bird and Flower market in Qingshanhu district was a large wholesale market; the others were all retail markets. Air samples were collected monthly from April 2014 to March 2015 via special air pumps (SQC-1000, Yancheng Tianyue Instrument & Meter Co., Ltd., Jiangsu, China). This equipment is widely applied in collecting aerial particulates and aerosols in China and uses a collection bottle (Qingdao Junray Intelligent Instrument Co., Ltd.) to collect air samples by passing through a transport medium. In each month, 10 air samples were collected in Jingdong Bird and Flower market, and 6 air samples were collected in each of the other retail LPMs. For retail markets, first, the wind direction in the market was determined. The poultry stall was chosen as the study center; 2 air samples were collected on the left and right of the stall at 3 m from the stall; and three other air samples were collected at 3 m, 5 m and 7 m from the stall in the downwind direction; another air sample was collected in the upwind direction at 1 m distance from the stall (Fig. 2a). For Jingdong Bird and Flower wholesale market, five air samples were collected in the east, south, west, and north and the center of the LPM. Samples were also collected 1 m, 3 m, 5 m and 7 m from the outside entrance (Fig. 2b). Each air sample was collected for 1.5 h, then stored in MEM (Gibco), aliquoted into 3 tubes and stored at −80 °C. To avoid excess bubbles, no BSA was included in the transport medium during sample collection; instead, BSA was added immediately after collection. All samples were transported on ice.

### Detection of influenza A nucleic acid and virus isolation

RNA was extracted with Qiagen RNeasy Mini kit, and influenza A was detected by RRT-PCR with primer and probe sets recommended by WHO (FluA-Forward: GACCRATCCTGTCACCTCTGAC; FluA-Reverse: GGGCATTYTGGACAAAKCGTCTACG;

FluA-probe: TGCAGTCCTCGCTCACTGGGCACG). If the aerosol samples were Flu A-positive, they were treated with 6 antibiotics (Penicillin: 2 × 10^6^ U/L, Streptomycin: 200 mg/L, Polymyxin: 2 × 10^6^ U/L, Gentamicin: 250 mg/L, Nystatin: 0.5 × 10^6^ U/L, Levofloxacin Hydrochloride: 600 mg/L) for 1 h, then virus isolation was conducted by inoculating the treated samples into 9- or 10-day-old specific-pathogen-free embryonated eggs. After incubation at 37 °C for 48 h, the allantoic liquid of the eggs was harvested and tested by Flu A fast diagnostic kits (Colloidal Gold Antibody Conjugates, Beijing NBGen Bio-technology Limited Company). If the result was positive, the allantoic liquid was aliquoted and stored at −80 °C.

### Whole-genome sequencing of influenza virus with MiSeq NGS platform

Viral RNA was extracted from harvested allantoic liquids using the Qiagen RNeasy Mini kit. Invitrogen SuperScript III One-step RT-PCR platinum Taq HiFi kit was used to amplify the RNA with one-step RT-PCR primers as follows: influenza primer A, GGGGGGAGCAAAAGCAGG; influenza primer B, GGGGGGAGCGAAAGCAGG influenza primer C, CGGGTTATTAGTAGAAACAAGG [14]. PCR products were purified by QIAGEN MinElute Reaction Cleanup Kit. One ng of DNA product was processed for NGS (next generation sequencing) sample preparation with the Illumina Nextera XT DNA Sample Preparation Kit (96 Samples), and sequencing was performed using a MiSeq v2 kit (500 cycles) to produce 2 × 250 paired-end reads (Illumina, San Diego, CA, USA) [15]. After automated cluster generation with MiSeq, the sequencing files were processed and genomic sequence reads were obtained. The full genome sequence was assembled by CLC platform.

### Phylogenetic analysis

Alignment of each segment was performed with the Mega (Version 6.05) software package; the phylogenetic trees of all the 8 segments were conducted by using neighbor-joining method with Kimura two-parameter distances. The bootstrap value was 1000.

## Results

### Sample collection and virus isolation

In total, 807 air samples were collected from 46 stalls in 10 different LPMs, from 4 districts in Nanchang city (Fig. 1) between April 2014 and March 2015. Of the 807 air samples, 275 samples (34.1%) were tested to be Flu A positive based on RRT-PCR results. Further statistical analysis suggested that air samples collected 3 m from poultry stalls were significantly more likely to test positive for Flu A than air samples collected at 5 and 7 m in the retail LPM (χ^2^ = 39.792, *p* < 0.01), while no difference was detected between the positive rate of air samples collected at 5 and 7 m (χ^2^ = 3.030, *p* > 0.05). In the wholesale LPM, the Flu A positive rates of air samples collected at 5 and 7 m were not significantly different (χ^2^ = 3.155, *p* > 0.05). The control samples were all negative for Flu A. One virus was successfully isolated from these Flu A RRT-PCR positive samples by inoculating samples into Specific Pathogen-Free (SPF) embryonated chicken eggs, and the HA titer of this virus was 256. This virus isolation positive sample was collected from Jingdong Bird and Flower market in Qingshanhu district, Nanchang city on July 31, 2014. Full genome sequencing of the virus showed it was a subtype-H9N2 virus and was termed as A/environment/Jiangxi/14737/2014 (H9N2) (Abbreviated as JX14737 here after). The sequences of all eight segments of this virus were submitted to the Global Initiative on Sharing All Influenza Data (GISAID) with accession numbers from EPI858159 to EPI858166.

### Phylogenetic analysis

**Fig. 3:**
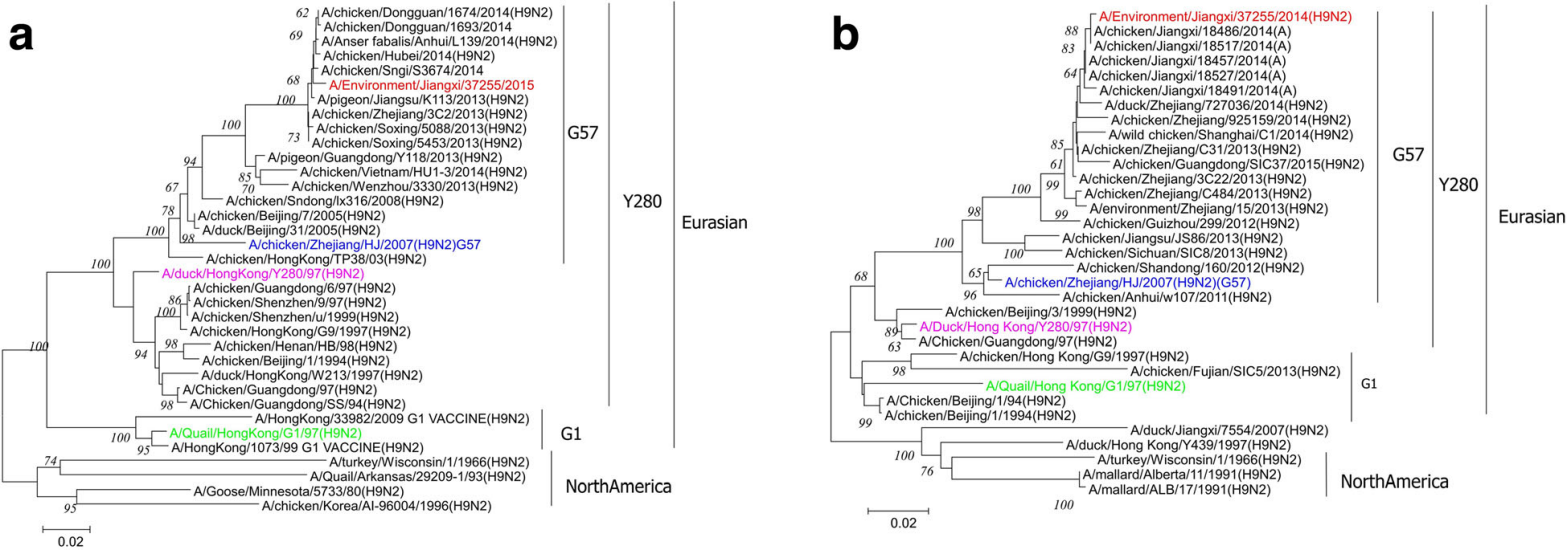
Phylogenetic trees of the HA and NA genes of JX14737. Trees were built by the neighbor-joining method using the MEGA6 software package (bootstrap value = 1000). The JX14737 H9N2 virus isolated from Jingdong Bird and Flower LPM in this study is highlighted by a *red circle*. The G57-like strain represented by A/chicken/Zhejiang/HJ/2007(H9N2) is highlighted by a *blue square*. The Y280-like strain represented by A/duck/Hong Kong/Y280/1997(H9N2) is highlighted by a *pink rhombus*. The G1-like strain represented by A/Quail/Hong Kong/G1/1997(H9N2) is highlighted by a *green triangle*. The phylogenetic trees of the HA and NA genes of JX14737 are **a** and **b**, respectively

To understand the genetic relationships between this aerosolized H9N2 virus and previously published H9N2 viruses, the homology of JX14737 with other viruses was analyzed with BLAST in GenBank, and it was found that JX14737 shared high nucleotide homology with the H9N2 AIVs from Jiangxi, Guangdong, Zhejiang, Anhui and Shanghai in China. The phylogenetic analysis of the HA and NA genes of the H9N2-subtype virus indicated that both HA and NA genes (Fig. 3a and b) belonged to a Eurasian avian lineage, and the virus fell into the G57 genotype cluster, which belonged to the Y280 lineage. The phylogenetic analysis of six internal genes of the H9N2-subtype virus showed that all of them fell into the G57 genotype cluster (Additional file 1A-F).

### Molecular characterization of the aerosolized H9N2 virus

A mutation at Q226L (H3 numbering) was detected in the HA protein, which indicated that the virus was prone to binding to α2,6-sialic acid receptor; this human-like receptor binding preference increased the risk of human infection of AIVs [16]. We downloaded 273 HA genes from GISAID of H9N2-subtype viruses that had been collected from different parts of the word between April 2014 and March 2015. After comparing JX14737 with these H9N2 sequences from GISAID, we found an unusual mutation in position 137 of the HA gene. Of these 273 sequences, most had K/T/S/N in position 137, with only 3 isolates showing A as in JX14737. Position 137 K was associated with avian-like receptor binding preference [17], but the significance of 137A had not been determined yet. The I292 V mutation in PB2 was associated with increased polymerase activity in 293 T cells, and the A588 V mutation enhanced polymerase activity, viral replication capability in mammalian and avian cells, and virulence in mice [18]. The hemagglutinin cleavage site of JX14737 possessed only one basic amino acid (PSRSSR↓GL) between HA1 and HA2, which indicated that this virus was a low pathogenic AIV in poultry (Table 1).

**Table 1:** Key amino-acid changes in the full genome of JX14737 virus isolated from air samples

Protein	Amino-Acid Mutations	Phenotype
PB2	292 V	Increases polymerase activity in 293 T cells
	588 V	Enhanced polymerase activity and viral replication in human NHBE cells
HA	Q226L^a^	Increased viral binding to α2,6-sialic acid receptor, airborne transmission between mammals
	N/K137A^a^	Avian-like receptor binding preference (N/137 K)

^a^The amino acid positions of HA gene represent H3 numbering

## Discussion

Subtype-H9N2 influenza virus was first identified in chicken farms in China in 1994 [19] and was found to be widespread in poultry and wild birds in Asia. Three major lineages of H9N2 virus circulated in poultry and wild birds: G1, Y280/G9, and BJ/94, represented by A/quail/Hong Kong/G1/97, A/duck/Hong Kong/Y280/97, and A/chicken/Beijing/1/1994 [20], respectively. H9N2 virus has been a gene donor for internal segments to many novel AIVs. Recently, many human infections with novel AIVs have occurred in China [3, 12, 21], especially in southern and eastern China. For instance, human infections with novel H7N9 and H10N8 AIVs were first reported in eastern China in 2013. Analysis of the full genomes of H7N9 and H10N8 viruses isolated from the patients showed that all 6 internal segments of these virus had originated in H9N2 AIVs [2, 4]. Since 2015, recent human infections with the high pathogenic avian influenza virus H5N6 have also been reported to have acquired internal genes from H9N2, although the first H5N6 virus found in humans had internal genes from H5N1 [22].

Sporadic human infections with H9N2 virus have been reported since the 1990s. Epidemiology data showed that some of these patients did not have direct contact with live poultry or any history of live poultry exposure [3]. These studies suggested that humans could be infected by AIVs without contacting live poultry directly. In this study, monthly air sample surveillance for AIV was conducted at 46 sites in 10 LPMs in Nanchang between April 2014 and March 2015. Of 807 air samples, 275 samples (34.1%) were Flu A positive by RRT-PCR, indicating a relatively high proportion of nucleic acid contamination in the air of LPMs. In addition, one (0.124%) H9N2 virus was isolated in the Jingdong bird and flower market, a large wholesale market in Nanchang city, Jiangxi province. The virus isolated from air samples was a low pathogenic AIV, but some mammalian-adaptation mutations were detected, along with mutations relating to polymerase activity and virus replication. Airborne virus has also been detected in LPMs in Guangdong [9] and Hong Kong [12] in South China, both of which are in or adjacent to tropical areas, while in this study it was found in Jiangxi province, a typical subtropical area in central China.

There were some limitations to this study. First, the air sample collection time (1.5 h) and the distance between stall center and instrument might affect the results. Second, the presence of polluted particles in the air might decrease the sensitivity rate of virus isolation.

## Conclusions

In our study, one H9N2 AIV was successfully isolated from 807 aerosolized samples. The virus shared high nucleotide homology with H9N2 AIVs from South China. Our study provided further evidence that the air in LPMs can be contaminated by influenza virus and its nucleic acids, and this fact should be considered when choosing and evaluating possible disinfection strategies for LPMs, such as regular air disinfection. Further statistical analysis suggested that the Flu A positive rate of air samples collected 3 m from poultry stalls were significantly higher than that of air samples collected at 5 and 7 m in the retail LPM (χ^2^ = 39.792, *p* < 0.01). The results suggested that the risk of human infection by aerosolized virus was increased when people were closer to stalls selling live poultry.

## Additional file

Additional file 1: Figure S1. Phylogenetic trees of the internal genes of JX14737. Trees were built by the neighbor-joining method using the MEGA6 software package (bootstrap value = 1000). The JX14737 H9N2 virus isolated from Jingdong Bird and Flower LPM in this study is highlighted by a red circle. The G57-like strain represented by A/chicken/Zhejiang/HJ/2007(H9N2) is highlighted by a blue square. The Y280-like strain represented by A/duck/Hong Kong/Y280/1997(H9N2) is highlighted by a pink rhombus. The G1-like strain represented by A/Quail/Hong Kong/G1/1997(H9N2) is highlighted by a green triangle. The phylogenetic trees of PB2, PB1, PA, NP, M, and NS genes of JX14737 are a-f, respectively. (PDF 343 kb)

## Abbreviations

AIVs: Avian Influenza Virus
GISAID: Global Initiative on Sharing All Influenza Data
LPMs: Live poultry markets
NGS: Next generation sequencing
RRT-PCR: Real-Time Reverse Transcription-Polymerase Chain Reaction
