# Supplementary figures and images for: Avian influenza H9N2 virus isolated from air samples in LPMs in Jiangxi, China
Source: Virol J. 2017 Jul 24;14:136. doi: 10.1186/s12985-017-0800-y (PMC5525224; doi:10.1186/s12985-017-0800-y)

a

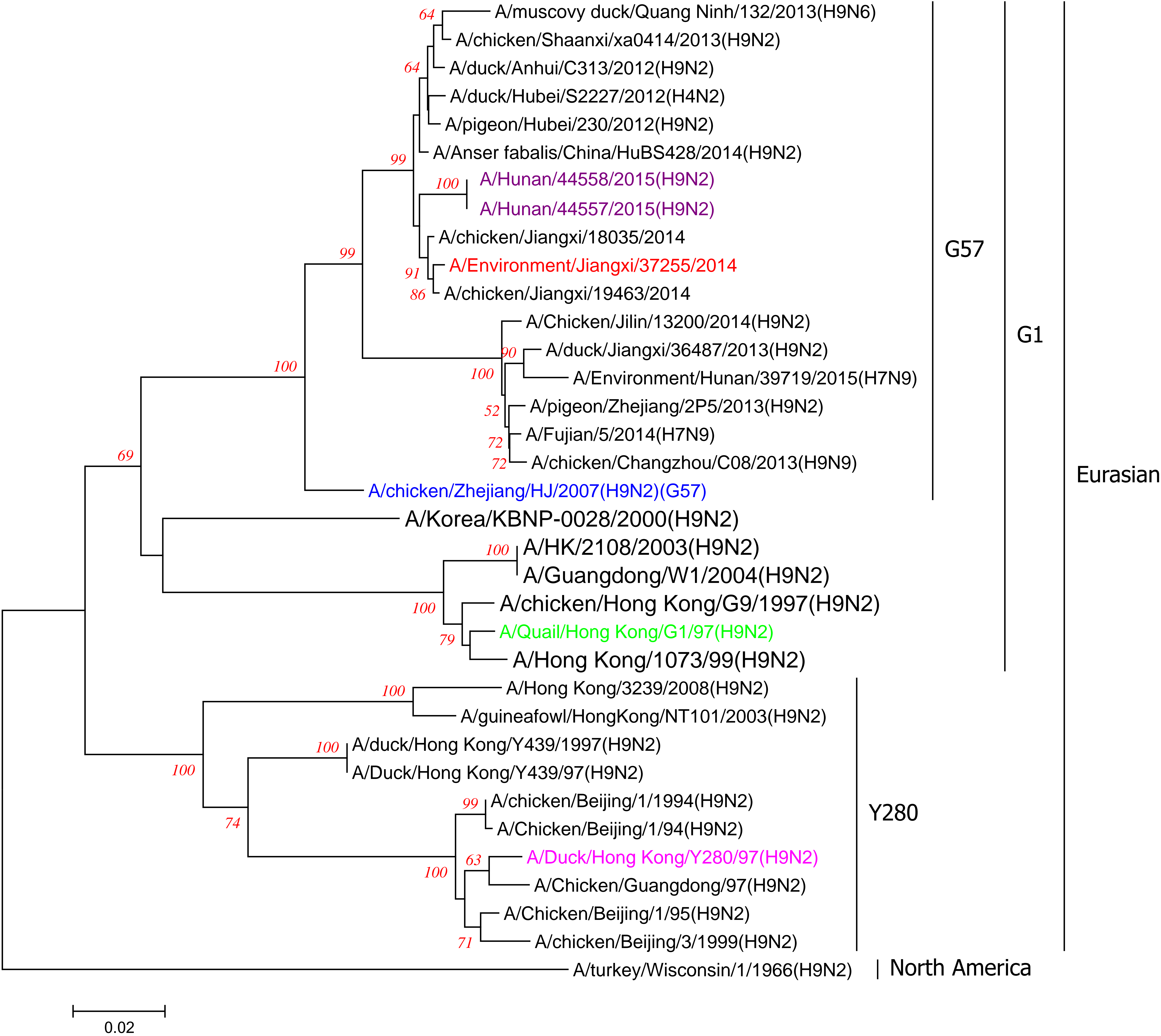

b

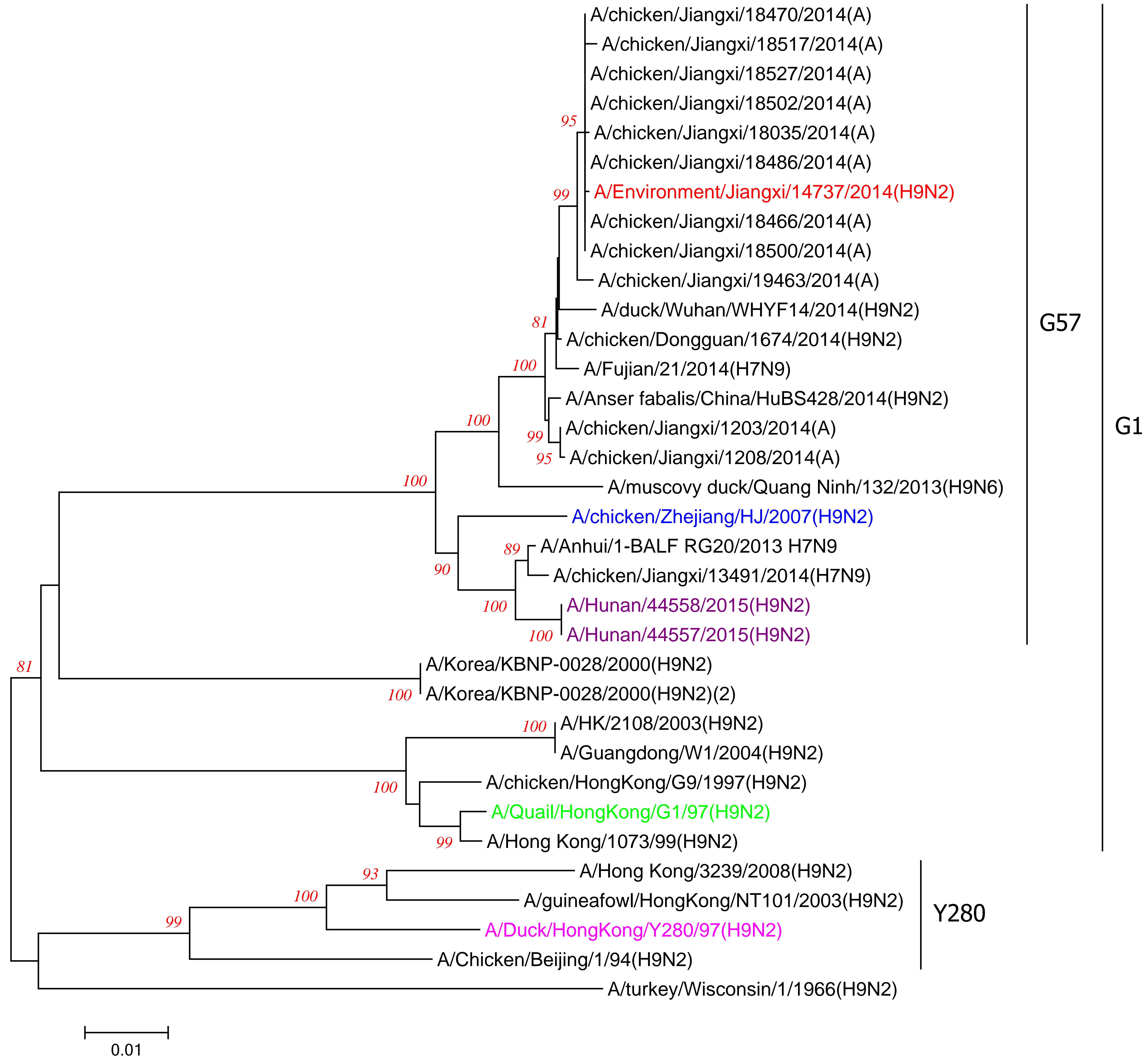

C

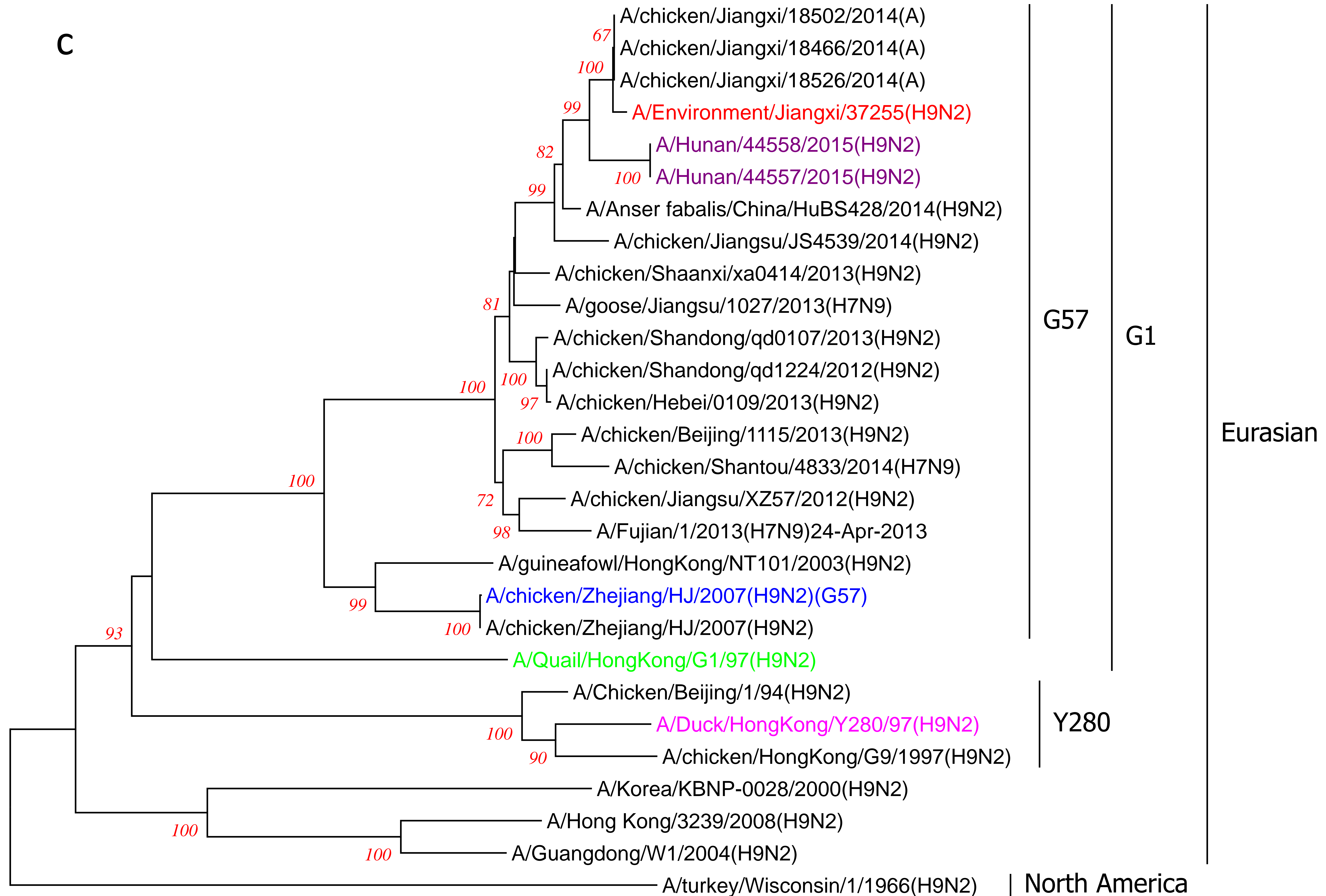

0.02

d

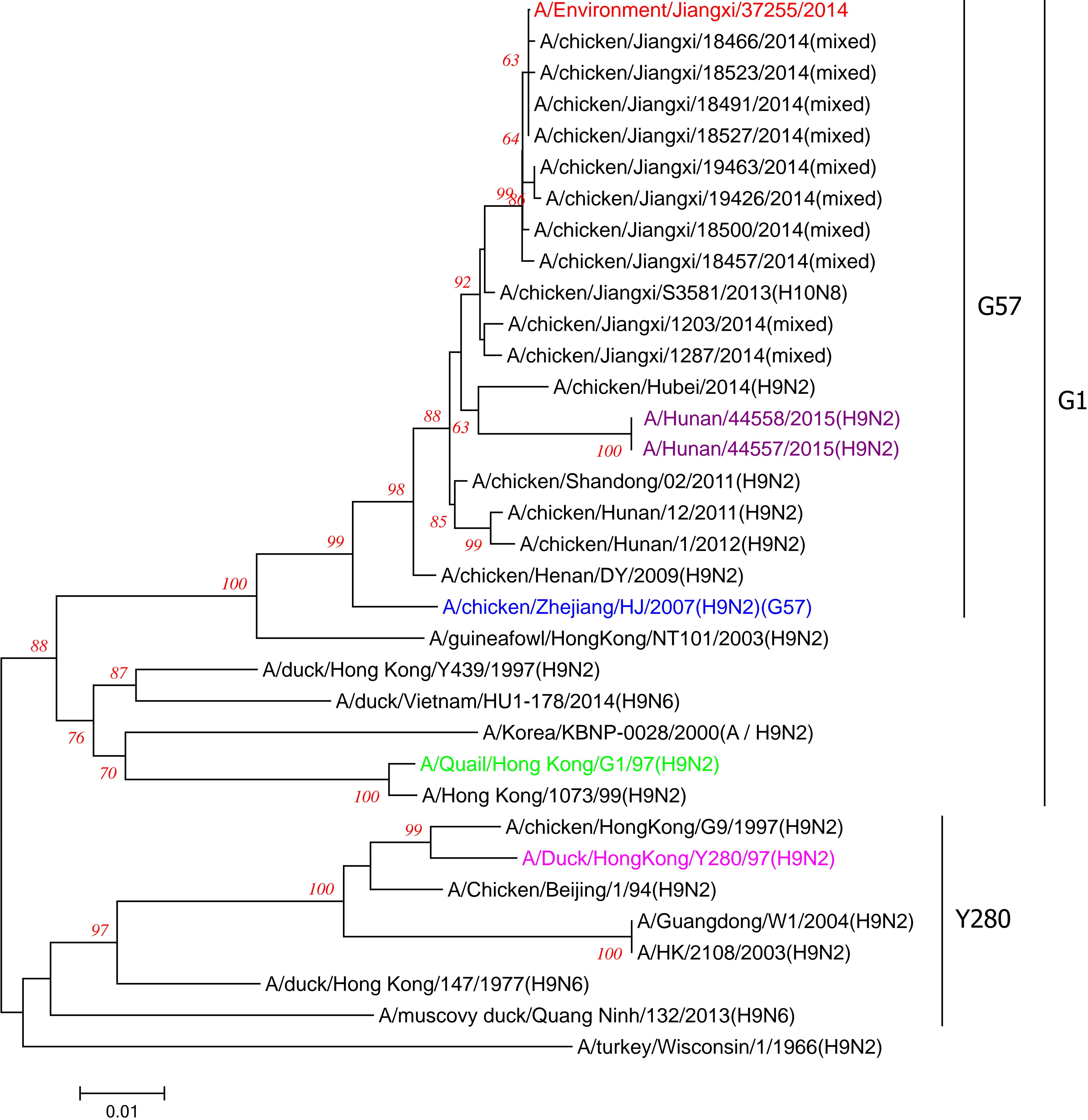

e

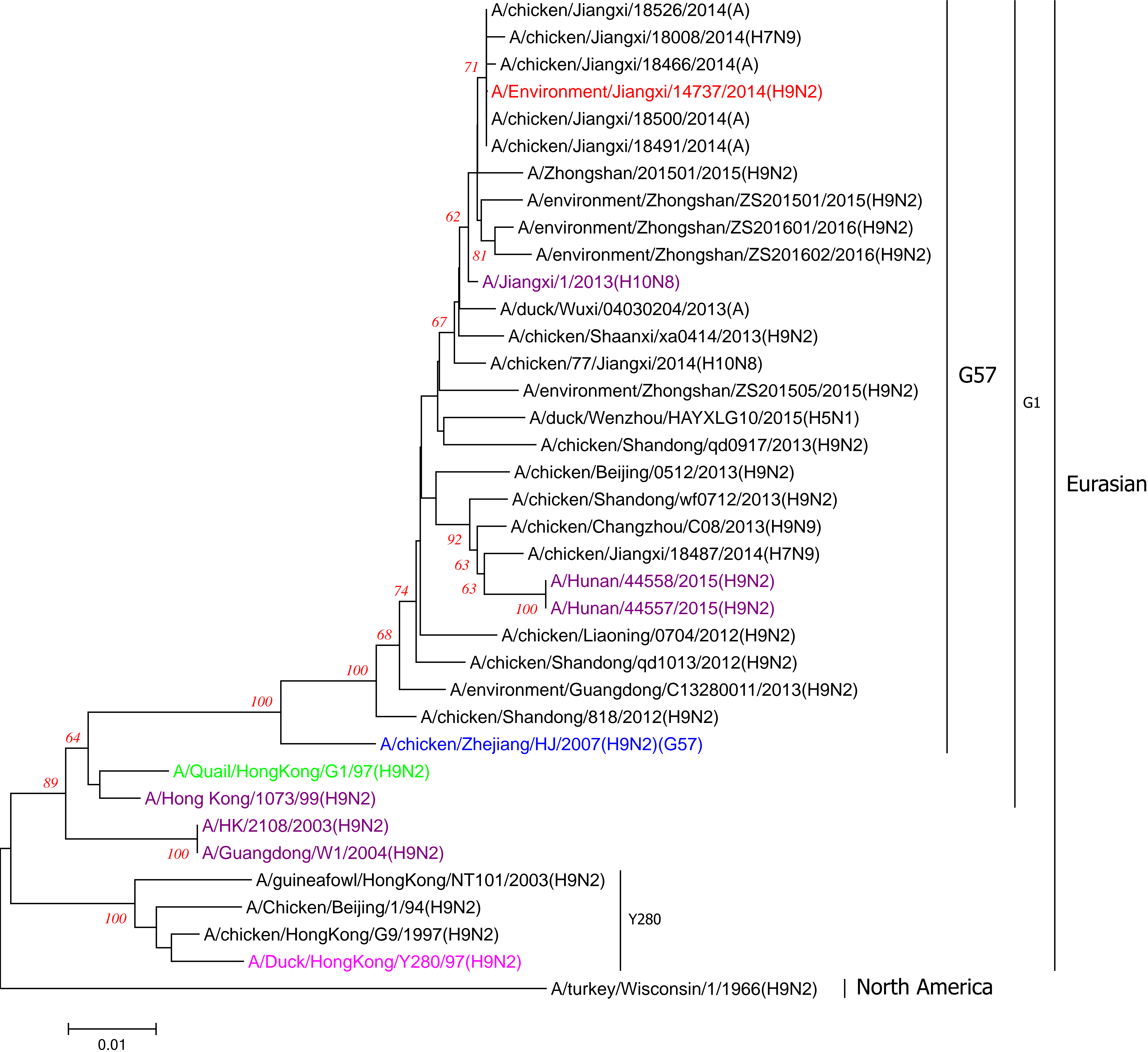

f

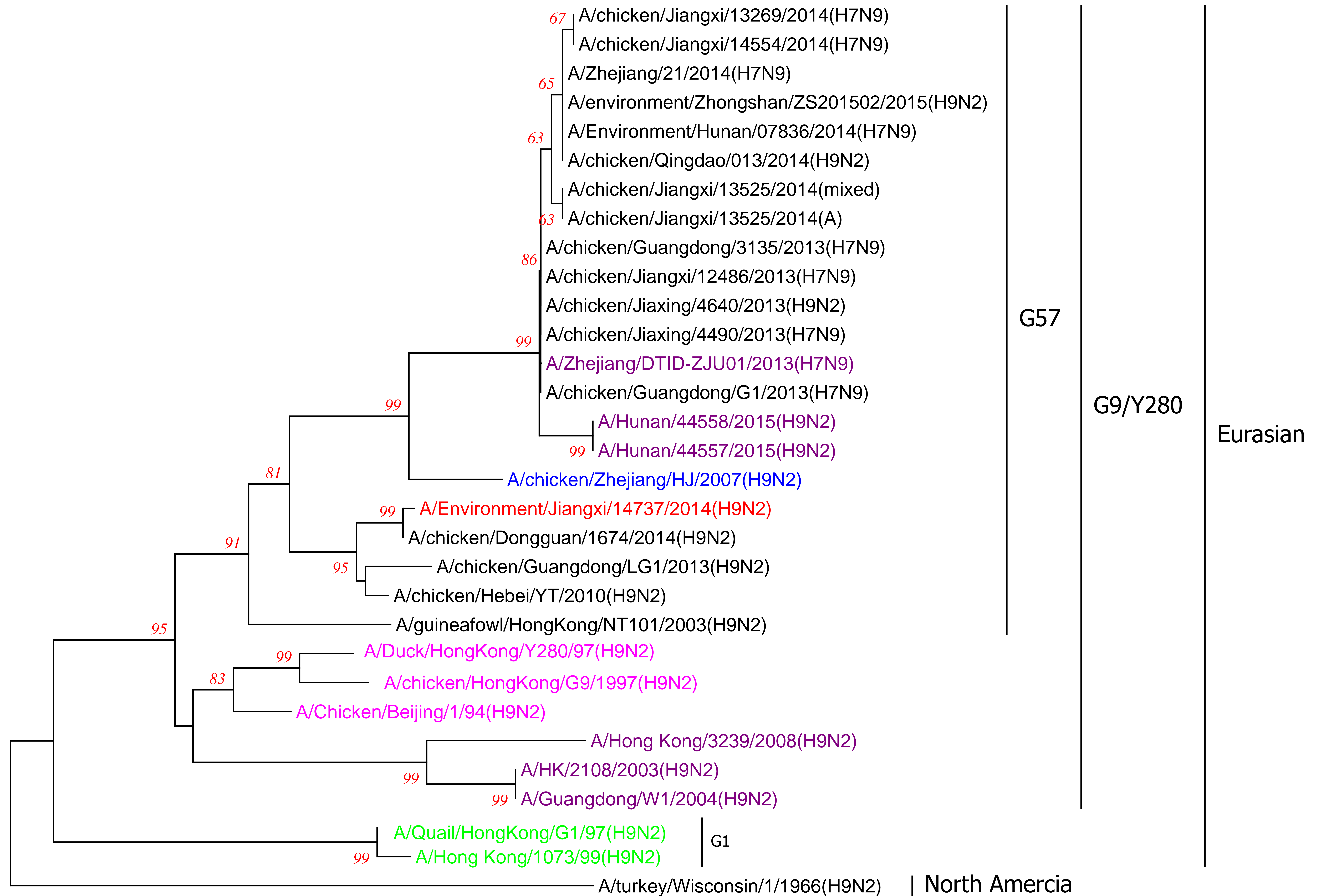

Supplement: Additional file 1: Figure S1. — Phylogenetic trees of the internal genes of JX14737. Trees were built by the neighbor-joining method using the MEGA6 software package (bootstrap value = 1000). The JX14737 H9N2 virus isolated from Jingdong Bird and Flower LPM in this study is highlighted by a red circle. The G57-like strain represented by A/chicken/Zhejiang/HJ/2007(H9N2) is highlighted by a blue square. The Y280-like strain represented by A/duck/Hong Kong/Y280/1997(H9N2) is highlighted by a pink rhombus. The G1-like strain represented by A/Quail/Hong Kong/G1/1997(H9N2) is highlighted by a green triangle. The phylogenetic trees of PB2, PB1, PA, NP, M, and NS genes of JX14737 are a-f, respectively. (PDF 343 kb) [file 12985_2017_800_MOESM1_ESM.pdf]
